# Changes in cannabis use, exposure, and health perceptions following legalization of adult recreational cannabis use in California: a prospective observational study

**DOI:** 10.1186/s13011-021-00352-3

**Published:** 2021-02-12

**Authors:** Kathleen Gali, Sandra J. Winter, Naina J. Ahuja, Erica Frank, Judith J. Prochaska

**Affiliations:** 1 Department of Medicine, Stanford Prevention Research Center, Stanford University, Medical School Office Building, X316, 1265 Welch Road, Stanford, CA 94305-5411 USA; 2 Faculty of Medicine, University of British Columbia, Vancouver, BC Canada

**Keywords:** Cannabis, Legalization, Marijuana, Perceptions

## Abstract

**Background:**

Most U.S. states have legalized cannabis for medical and/or recreational use. In a 6-month prospective observational study, we examined changes in adult cannabis use patterns and health perceptions following broadened legalization of cannabis use from medical to recreational purposes in California.

**Methods:**

Respondents were part of Stanford University’s WELL for Life registry, an online adult cohort concentrated in Northern California. Surveys were administered online in the 10 days prior to state legalization of recreational use (1/1/18) and 1-month (2/1/18–2/15/18) and 6-months (7/1/2018–7/15/18) following the change in state policy. Online surveys assessed self-reported past 30-day cannabis use, exposure to others’ cannabis use, and health perceptions of cannabis use. Logistic regression models and generalized estimating equations (GEE) examined associations between participant characteristics and cannabis use pre- to 1-month and 6-months post-legalization.

**Results:**

The sample (*N* = 429, 51% female, 55% non-Hispanic White, age mean = 56 ± 14.6) voted 58% in favor of state legalization of recreational cannabis use, with 26% opposed, and 16% abstained. Cannabis use in the past 30-days significantly increased from pre-legalization (17%) to 1-month post-legalization (21%; odds ratio (OR) = 1.28, *p*-value (*p*) = .01) and stayed elevated over pre-legalization levels at 6-months post-legalization (20%; OR = 1.28, *p* = .01). Exposure to others’ cannabis use in the past 30 days did not change significantly over time: 41% pre-legalization, 44% 1-month post-legalization (OR = 1.18, *p* = .11), and 42% 6-months post-legalization (OR = 1.08, *p* = .61). Perceptions of health benefits of cannabis use increased from pre-legalization to 6-months post-legalization (OR = 1.19, *p* = .02). Younger adults, those with fewer years of education, and those reporting histories of depression were more likely to report recent cannabis use pre- and post-legalization. Other mental illness was associated with cannabis use at post-legalization only. In a multivariate GEE adjusted for sociodemographic characteristics and diagnoses, favoring legalization and the interaction of time and positive health perceptions were associated with a greater likelihood of using cannabis.

**Conclusions:**

Legalized recreational cannabis use was associated with greater self-reported past 30-day use post-legalization, and with more-positive health perceptions of cannabis use. Future research is needed to examine longer-term perceptions and behavioral patterns following legalization of recreational cannabis use, especially among those with mental illness.

## Background

In the United States, a majority of states have legalized the use of cannabis for some purposes, and California has been a forerunner in cannabis legalization. California was the first state to legalize medical cannabis use in 1996. To date, 35 states (plus the District of Columbia, Guam, and Puerto Rico) have legalized medical cannabis; 15 of these 35 (plus the District of Columbia and Guam) have also legalized adult recreational cannabis, and another 13 states permit the use of products with low-tetrahydrocannabinol (THC, cannabis’s primary psychoactive ingredient) for medical purposes [1].

Modes of cannabis use include smoking, vaping, and as an edible. Cannabis when smoked may be wrapped in paper (i.e., a joint) or placed within a hollowed-out cigar (i.e., a blunt). In 2018, modes of recent cannabis use among young adults (18–25 years old) in California were reported as 81% smoking (i.e., joint, bong, pipe); 47% vaping; 43% blunt use; and 35% eating/drinking; 78% reported more than one method [2].

Potential mental health harms of cannabis use include increased risk of developing schizophrenia and other psychoses, with heavier cannabis use associated with greater risk [3]. Depression, anxiety, and suicidal thoughts also have been linked to cannabis use [4]. Whether the associations are causal is unknown. Smoking cannabis can affect lung health, with regular use associated with chronic bronchitis [3]. Smoking cannabis during pregnancy is associated with low birthweight; studies of adverse effects of prenatal cannabis use on offspring behavior and cognitive development have been equivocal [5]. Studies of brain structural measures and cannabis use in youth and young adults also have produced mixed results [6].

To characterize the evidence on the health benefits of cannabis use, the National Academies of Sciences, Engineering, and Medicine published a comprehensive in-depth review of 10,000 studies [3]. The report found strong evidence from randomized control trials to support the conclusions that cannabis or its constituents (i.e., cannabinoids) are effective for treating chronic pain; as antiemetics in the treatment of chemotherapy-induced nausea and vomiting; and for improving patient-reported multiple sclerosis spasticity symptoms. With regards to mental health, other research has found an anxiolytic-like effect of cannabidiol (CBD) in patients with social anxiety disorder [7]. There also is moderate evidence for cannabinoids, mainly nabiximols, in improving short-term sleep outcomes in those with chronic medical conditions (e.g., fibromyalgia) [8]. Few studies have examined cannabis’s effects on well-being, a construct related to quality of life, and findings have been mixed [9, 10].

Since 1990, there has been an increasing trend in favor of legalizing cannabis in the United States. The Pew Research Center reported that 59% of Americans favor legalizing cannabis for medical and recreational use, while another 32% support cannabis use for medical purposes only; only 8% opposed legalizing cannabis [11]. Since 2002, adult use of cannabis has been increasing. In 2019, 31.6 million Americans reported cannabis use in the past 30 days, with prevalence of 23% among adults aged 18-to-25 and 10.2% among adults 26 years and older [12]. Data from the 2018 California Health Interview Survey showed that among adults 18 years and older, 33% reported cannabis use within the past month [13].

In California, on November 8, 2016, voters passed Prop 64, the Adult Use of Marijuana Act, supporting the legalization of recreational cannabis use for adults 21 years or older. Prop 64 proposed to create a system to regulate the cannabis market and impose taxes on the retail sale and cultivation of cannabis; and allowed for use in a private home or at a business licensed for on-site consumption, and prohibited use while driving and in public areas including federal areas such as parks, as it is illegal under federal law [14]. On January 1, 2018, California was authorized to begin issuing licenses to operate recreational cannabis businesses, legalizing sales from licensed retail outlets and the purchasing of cannabis for recreational use [15].

States that legalized recreational cannabis use had a higher prevalence of cannabis use and greater use of products such as cannabis edibles, drinks, and high potency concentrate than in those that had not [16]. Among the first four states that legalized cannabis for recreational purposes (Colorado, Washington, Alaska, and Oregon), there were increases in frequent cannabis use (defined as use 20 days or more in the past month) and cannabis use disorder among adults aged 26 and older following recreational cannabis legalization [17]. A recent California study found no increase in cannabis use after legalization of recreational cannabis; however, the study sample was restricted to young adults aged 18–24 who used tobacco, so the findings may not generalize to the broader population [18]. Beliefs on the health benefits of cannabis (i.e., for pain management, relief from stress) was found to be higher in states that had legalized cannabis for recreational use [19]. With expanding legalization and increases in cannabis use, examining patterns of cannabis use and the factors that might drive cannabis use trends over time is needed.

With California’s legalization of recreational cannabis use, we sought to characterize, from pre- to post-policy implementation, adults’ use patterns, exposure to others’ cannabis use, and perceptions of the benefits or harms of cannabis use to physical and mental health and well-being. We hypothesized an increase in adult cannabis use and exposure to others’ use as well as more positive health perceptions of cannabis use over time.

## Methods

### Study design

California’s broadening of cannabis legalization from medical-only to also include adult recreational use was effective January 1, 2018. In the 10 days prior to the date of state legalization of recreational use (12/21/17–12/31/17), respondents were recruited online from Stanford University’s WELL for Life registry, an online adult cohort concentrated in Northern California, with the mission to accelerate the science to enhance health and well-being. Respondents gave informed consent and were re-assessed 1 month (2/1/18–2/15/18) and 6 months (7/1/18–7/15/18) following the change in state policy. All research activities were approved by Stanford’s Institutional Review Board.

### Measures

All measures were completed online with REDCap [20].

#### Demographic characteristics

Respondents self-reported their gender, age, race/ethnicity, years of education, employment status (employed, home-maker, student, disabled, temporarily laid off, unemployed, retired, other), and marital status (married, living with a partner or significant other, divorced, separated, widowed, single). Gender was coded for analyses as male/trans male and female. Race/ethnicity was coded for analyses as non-Hispanic White and other.

#### Support for legalizing recreational cannabis use

Policy support was assessed at pre-legalization as “Did you vote in favor or opposition to California Proposition 64 to legalize recreational use of marijuana in the state for persons aged 21 or older?” with response options of “vote in favor”, “vote in opposition”, or “did not vote.”

#### Cannabis use and exposure to Other’s use

Self-reported cannabis use, mode(s) of use during the past 30 days (i.e., smoked, vaporized, edible, in a blunt) and most recent exposure to others’ cannabis use (today, yesterday, last 3 to 7 days [this week], last 8 to 14 days [last week], last 15 to 30 days [past month], more than a month ago but within the past year, more than a year ago, never) were assessed. Use and exposure variables were dichotomized to reflect past 30-day cannabis use and past 30-day exposure to others’ cannabis use (yes: within the past 30 days, no: greater than 30 days) for analyses.

#### Health perceptions

Perceptions of cannabis’ effects were assessed with three questions with the same stem: “How harmful or beneficial do you think marijuana is to … physical health / mental health / well-being?” with response options ranging from extremely harmful (− 4) to neither harmful nor beneficial (0) to extremely beneficial (+ 4), with values and anchors of less degree of harm/benefit in between. Participants could also mark “don’t know” and “refused.” “Don’t know” was coded as a 0 and “refused” was coded as missing. The three perception items were averaged to produce a single scale score for analyses with Cronbach alpha = 0.91.

#### Medical characteristics

Participants self-reported pain for the past 2 weeks (yes/no) and lifetime diagnoses of cancer, depression, and other mental illness (yes/no).

### Data analysis

Descriptive statistics were run on sample baseline characteristics. Surveys were completed by *N* = 429 at pre-legalization, *N* = 323 at 1-month post-legalization, and *N* = 225 at 6-months post-legalization, with attrition of 24.7% at 1-month and 47.6% at 6-months post-legalization. Compared to those who missed one or two surveys (*n* = 204, 47.6%), respondents who completed all three surveys (*n* = 225, 52.4%) were less likely to be employed (odds ratio (OR) = 0.55, *p*-value (*p*) = .02). None of the other measured variables predicted attrition (all *p*-values > 0.06). Employment was included as a covariate in all longitudinal models.

Past 30-day cannabis use and exposure to others’ cannabis use were calculated as frequencies and then also modeled over time using generalized estimating equations (GEE) [21], which included all available data, modeled missing responses, and adjusted for variables related to attrition. GEE models also were run to examine cannabis-related health perceptions over time. Linear and binary GEE models were used for continuous and binary outcome variables, respectively.

To examine predictors of cannabis use, initial univariate logistic regression models were run to identify respondent characteristics associated with cannabis use at each time point. Next, a series of multivariate GEE models were run. The initial GEE model controlled for variables related to attrition. The second GEE model added demographic variables significantly associated with cannabis use at any time point at *p* < .05. The final GEE model added in vote, health perceptions, and 2-way interaction terms for age and health perceptions with time. Interaction terms with *p* < .10 were dropped from the model. All GEE models used an unstructured correlation structure. Analyses were conducted using SPSS version 25 [22].

## Results

### Sample description

The sample (*N* = 429) had a mean age of 56 years (standard deviation (SD) = 14.6, range: 23 to 86); 51.3% identified as female; 55.5% identified as non-Hispanic White/Caucasian; 41.0% were married/cohabitating, 8.2% were divorced/separated, 11.4% were never married, and 2.3% were widowed; 46.6% were employed, 13.5% were retired, and 6.8% were other (e.g., student, unemployed). Years of education ranged from 12 to 20 with a mean of 17.3 years (SD = 2.0). Over half (58.0%) of the sample reported voting in favor of state legalization of recreational cannabis use, 25.9% opposed, and 16.1% abstained. See Table 1.

**Table 1:** Sample Characteristics (*N* = 429)

Variables	*N*	Percent
Sex		
Male	83	19.3%
Female	220	51.3%
Trans Male	1	0.2%
Missing	125	29.1%
Age, Mean (SD)	56.0	(14.6)
Missing	146	34.0%
Race/Ethnicity		
Non-Hispanic White	238	55.5%
Hispanic	18	4.2%
Other	82	19.1%
Missing	91	21.2%
Marital status		
Married	156	36.4%
Living with significant other	20	4.7%
Divorced	32	7.5%
Separated	3	0.7%
Widowed	10	2.3%
Single	49	11.4%
Missing	159	37.1%
Employment status		
Currently employed	200	46.6%
Homemaker	8	1.9%
Student	3	0.7%
Disabled	2	0.5%
Unemployed	6	1.4%
Retired	58	13.5%
Other	10	2.3%
Missing	142	33.1%
Years of education, Mean (SD)	17.3	(2.0)
Missing	161	37.5%

### Cannabis use, exposure to others’ cannabis use, and mode of use

**Fig. 1:**
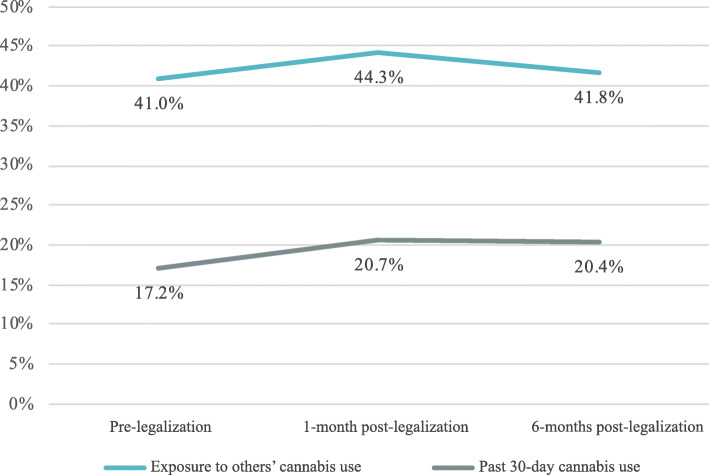
Cannabis use and exposure at pre- and 1-month post- and 6-months post-legalization. Exposure to other’s cannabis use increased from pre- to post-legalization but was not statistically significant. Past 30-day cannabis use significantly increased from pre-legalization to 1-month post-legalization and stayed significantly higher over pre-legalization levels at 6-months post-legalization

Figure 1 shows the prevalence of reported past 30-day cannabis use over time, which was 17.2% at pre-legalization, 20.7% at 1-month post-legalization, and 20.4% at 6-months post-legalization. In GEE models including all available data, modeling missing responses, and adjusting for employment status, cannabis use significantly increased from pre-legalization to 1-month post-legalization (OR = 1.28, *p* = .01) and stayed significantly higher over pre-legalization levels at 6-months post-legalization (OR = 1.28, *p* = .01). Among those reporting past 30-day cannabis use, the mean number of days used was 11 (SD = 9.4) at pre-legalization, 12 (SD = 10.4) at 1-month post-legalization, and 13 (SD = 11) at 6-months post-legalization.

Figure 1 also shows the prevalence of past 30-day exposure to others’ cannabis use, which was 41.0% at pre-legalization, 44.3% at 1-month post-legalization, and 41.8% at 6-months post-legalization. In GEE models including all available data, modeling missing responses, and adjusting for employment status, exposure to others’ cannabis use in the past 30 days did not change significantly from pre-legalization to 1-month post-legalization (OR = 1.18, *p* = .11) or 6-months post-legalization (OR = 1.08, *p* = .61).

**Fig. 2:**
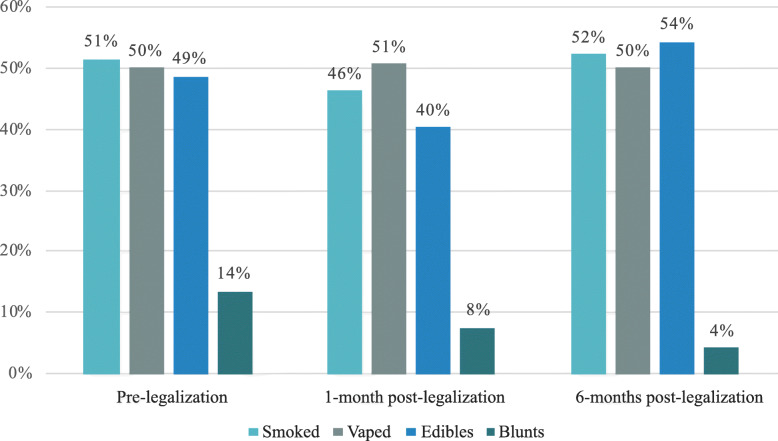
Reported modes of cannabis use over time among recent users. Most frequent modes of cannabis use were smoked, vaped, and edibles. Blunts were reported by a minority and declined over time

Figure 2 shows the modes of cannabis use over time among recent users, with multiple response options possible. Most frequent modes of cannabis use were smoked, vaped, and edibles. Blunts were reported by a minority and declined over time from 13.5% at pre-legalization to 4.3% at 6-months post-legalization.

### Health perceptions of cannabis over time

**Fig. 3:**
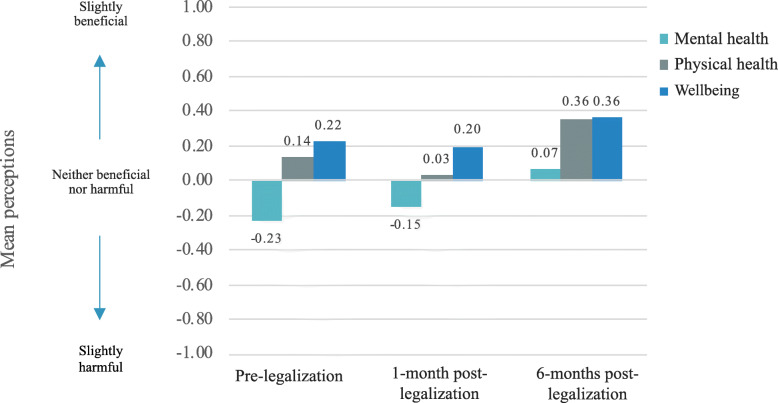
Mean perceptions over time. Perceptions of mental health, physical health, and wellbeing of cannabis use increased over time. Possible responses for each scale ranged from extremely harmful (− 4) to neither harmful nor beneficial (0) to extremely beneficial (+ 4), with values and anchors of less degree of harm/benefit in between

Figure 3 shows change over time in the sample’s mean health perceptions of cannabis. Mental health perceptions of cannabis use increased over time from slightly harmful at pre-legalization to slightly beneficial at 6-months post-legalization. Physical health perceptions of cannabis were positive overtime but decreased from pre-legalization to 1-month post-legalization, and then increased to levels above pre-legalization at 6-months-post-legalization. Well-being perceptions of cannabis use had the highest means overtime and remained similar at pre-legalization and 1-month post-legalization and then increased at 6-months post-legalization.

Estimated marginal means for the combined health perceptions of cannabis use item, with a potential range of − 4 to + 4, were 0.039 at pre-legalization, 0.005 at 1-month post-legalization, and 0.231 at 6-months post-legalization. That is, they were near zero at pre and 1-month post-legalization (i.e., neutral) and showed more positive health perceptions by 6-months post-legalization. In GEE models including all available data, modeling missing responses, and adjusting for employment status, cannabis use was perceived to be more beneficial at 6-months post-legalization than pre-legalization (OR = 1.19, *p* = .02), with no significant difference between pre-legalization and 1-month post-legalization (OR = 0.95, *p* = .40).

### Associations with cannabis use over time

Table 2 summarizes univariate associations between respondent characteristics and past 30-day cannabis use. At pre-legalization, correlates of recent cannabis use were younger age (OR = 0.98, 95% confidence interval (CI): 0.96, 0.999); less years of education (OR = 0.82, 95% CI: 0.70, 0.95); depression diagnosis (OR = 2.70, 95% CI: 1.38, 5.31); voting in favor of legalizing recreational cannabis use (OR = 8.74, 95% CI: 3.08, 24.81) or abstaining from voting (OR = 5.34, 95% CI: 1.62, 17.60) relative to those who opposed; and perceiving greater health benefits of cannabis (OR = 2.19, 95% CI: 1.80, 2.67).

**Table 2:** Univariate tests of associations with cannabis use at pre-legalization and 1- and 6-months post-legalization

	Cannabis use											
	Pre-legalization				1-month post-legalization				6-months post-legalization			
Variables	***n***	OR	95% CI	***p***	***n***	OR	95% CI	***p***	*n*	OR	95% CI	***p***
Female	278	1.11	0.56, 2.22	.77	269	0.90	0.48, 1.71	.76	183	0.83	0.38, 1.79	.63
Age (years)	**260**	**0.98**	**0.96, 0.999**	**.04**	**251**	**0.97**	**0.95, 0.99**	**.009**	**167**	**0.96**	**0.94, 0.99**	**.001**
Non-Hispanic White	309	0.56	0.31, 1.02	.06	301	0.74	0.41, 1.31	.30	201	0.77	0.38, 1.56	.47
Education (years)	**245**	**0.82**	**0.70, 0.95**	**.01**	**251**	**0.87**	**0.75, 0.999**	**.05**	169	0.85	0.72, 1.01	.07
Married/cohabitating	247	0.56	0.29, 1.07	.08	253	0.74	0.40, 1.37	.34	169	0.54	0.26, 1.12	.10
Currently employed	262	1.18	0.59, 2.39	.64	268	0.82	0.44, 1.53	.54	181	0.78	0.38, 1.62	.51
Medical condition												
Pain	243	0.90	0.43, 1.86	.78	249	0.69	0.34, 1.40	.30	166	0.56	0.24, 1.33	.19
Cancer	244	0.80	0.31, 2.05	.65	250	0.94	0.40, 2.18	.88	167	0.34	0.10, 1.21	.10
Depression	**242**	**2.70**	**1.38, 5.31**	**.004**	**248**	**2.29**	**1.20, 4.36**	**.01**	**166**	**3.00**	**1.40, 6.46**	**.005**
Other mental illness	242	1.99	0.85, 4.67	.12	248	1.56	0.64, 3.78	.33	**166**	**3.38**	**1.21, 9.49**	**.02**
Vote	391				302				202			
Oppose (ref)												
Abstain		**5.34**	**1.62, 17.60**	**<.01**		2.33	0.73, 7.45	.15		4.09	0.94, 17.83	.06
Favor		**8.74**	**3.08, 24.81**	**<.001**		**5.10**	**2.09, 12.45**	**<.001**		**6.74**	**1.97, 23.04**	**.002**
Health Perceptions^a^	**390**	**2.19**	**1.80, 2.67**	**<.001**	**300**	**2.34**	**1.86, 2.93**	**<.001**	**201**	**2.03**	**1.59, 2.59**	**<.001**

^a^ Health perceptions at pre-legalization

As seen at pre-legalization, a depression diagnosis had a 2- to 3-fold greater likelihood of cannabis use at 1- and 6-months post-legalization. Age and voting in favor of legalizing recreational cannabis use also remained associated with a greater likelihood of cannabis use at both post-legalization assessments. Less years of education was associated with a greater likelihood of cannabis use at 1-month but not 6-months post-legalization. Lastly, at 6-months post-legalization, other mental illness had a 3-fold greater likelihood of cannabis use.

In GEE analyses modeling cannabis use over time and adjusting for age, years of education, employment status, depression, and other mental illness, initial vote in favor of legalizing cannabis for recreational use (OR = 4.62, *p* < .01) and positive health perceptions (OR = 1.38, *p* < .001) remained significant correlates. There was a significant interaction effect of time at 6-months post-legalization and positive health perceptions (OR = 1.22, *p* = .01), suggesting that from pre-legalization to 6-months post-legalization, there was a greater increase in cannabis use among those with beneficial perceptions than those with harmful health perceptions of cannabis use. The interaction between health perceptions at 1-month post-legalization was not significant (OR = 1.08, *p* = .27) (Table 3). The interaction between time and age, was not significant (*p* > .19), and therefore was dropped from the model.

**Table 3:** Generalized estimating equation (GEE) models predicting cannabis use

	Model 1 (***N*** = 282)			Model 2 (***N*** = 252)			Model 3 (*N* = 252)		
Variables	AOR	95% CI	***p***	AOR	95% CI	***p***	AOR	95% CI	***p***
Time									
Pre-legalization (ref)									
1-month post-legalization	1.28	1.07, 1.53	0.01	1.23	1.02, 1.48	0.03	1.27	0.99, 1.63	0.06
6-months post-legalization	1.28	1.06, 1.55	0.01	1.18	0.98, 1.41	0.08	0.94	0.71, 1.23	0.63
Currently employed^a^	0.91	0.51, 1.64	0.76	0.53	0.25,1.13	0.10	0.52	0.25, 1.09	0.09
Age				0.97	0.94, 0.99	< 0.01	0.96	0.94, 0.98	0.01
Years of education				0.88	0.75, 1.02	0.08	0.86	0.73, 1.01	0.06
Depression				2.39	1.22, 4.68	0.01	1.90	0.92, 3.92	0.08
Other mental illness				1.08	0.42, 2.76	0.88	1.55	0.58, 4.15	0.38
Vote									
Oppose (ref)									
Abstain							1.57	0.41, 6.02	0.51
Favor							4.62	1.55, 13.8	< 0.01
Health Perceptions							1.38	1.16, 1.64	<.001
Time x health perceptions									
Pre-legalization x health perceptions (ref)									
1-month post-legalization x health perceptions							1.08	0.95, 1.23	0.27
6-months post-legalization x health perceptions							1.22	1.04, 1.42	0.01

Note. Adjusted odds ratios (AOR), confidence interval (CI), reference group (ref)

The age x time interaction was not significant and therefore dropped from the model

^a^ All models adjusted for employment status, due to its association with attrition

## Discussion

In this prospective observational study on cannabis policy changes in California, legalized recreational cannabis use was associated with greater self-reported past 30-day use one-month post-legalization, and in the univariate model, remained significantly higher at 6-months post-legalization. Compared to California state data, our study sample had a lower frequency of past 30-day cannabis use [13]. Likely related, the sample had more harmful perceptions of cannabis use: 43% of respondents at baseline and 42% at 1-month post-legalization perceived cannabis use to be harmful, whereas United States data from the Substance Abuse and Mental Health Services Administration estimated 12% of young adults 18 to 25 years old and 29% of adults 26 years or older perceived great risk from smoking cannabis monthly [23].

Surprisingly, exposure to others’ use of cannabis did not change from pre- to post-legalization of recreational cannabis use in our study timeframe; however, it is possible that exposure to cannabis smoke may have increased earlier among our study population. A California Department of Public Health survey found that the rate of cannabis exposure in 2016 among adults aged 18 to 64 years was 21.5%, and by 2018 had doubled to 40%, which is similar to our findings on cannabis exposure [24]. In late-2016, Prop 64 was voted on and approved by voters of California and could explain the increase of secondhand cannabis exposure from 2016 to 2018.

Significant correlates of cannabis use at all time points included depression diagnosis, while having an other mental illness diagnosis was significantly associated with cannabis use only 6-months post-legalization. A recent study found that from 2005 to 2017, the prevalence of cannabis use among people in the United States with depression was increasing and that those with depression experienced a rapid decrease in perception of risk of cannabis use [25]. While not tested in our study, it is possible those with depression or other mental illness experienced decreasing perceptions of risk of cannabis use. Despite the growing evidence of cannabis use as an effective treatment in chronic pain [3, 26] and its use in mitigating side effects of cancer therapies, our study showed that those with pain and those with a history of cancer were not significantly more likely to use cannabis.

Among our California study sample of adults between the ages of 23 to 86 with a mean age of 56 years, we found younger age associated with cannabis use pre- and post-legalization. These findings are consistent with previous national studies on adults in the United States where cannabis use decreased with increasing age [27].

By 6-months post-legalization, perceived health benefits of cannabis use significantly increased, and in the multivariate model, health perceptions were associated with cannabis use over time. Notably, perceptions of health benefits of cannabis use for mental health showed the largest increase. The literature suggests potential anxiolytic effects of CBD [7], but also points to the association of mental health harms with high-potency cannabis use [28]. Though our sample had an overall positive perception of cannabis use in benefiting physical health and wellbeing, there was no significant association between cannabis use with pain or cancer, common conditions for which cannabis has been used to treat. Mass marketing and health promotions from cannabis dispensaries also may have contributed to the increase in perceived health benefits of cannabis. Since the legalization of recreational cannabis in California, which includes the selling of cannabis, dispensary ads and mass marketing campaigns promoting uses of cannabis have proliferated [29, 30]. Endorsements from social media influencers and celebrities, may also be adding to overall positive perception of cannabis use. As a response to the mass marketing, Los Angeles and San Diego counties have proposed restrictions on where cannabis ads and billboards can be placed [31]. Much is to be learned on whether these restrictions will impact perceptions and use. To ensure health harms are not ignored, public health interventions such as educational programs and health communications are needed to increase awareness.

Study limitations include that the data were self-reported by a relatively small convenience sample, and thus may not be generalizable to other populations. The sample was more non-Hispanic White (55.5% vs 36.5%) than the general population in California [32]; however, a similar percentage of Californians voted in favor for Proposition 64 (58% vs 57.1%) [33]. Another limitation is the high level of missing demographic data and high attrition rate. Rather than remove respondents who did not complete all the surveys and conduct a complete-case analysis that could lead to less power and biased results, we used GEE analyses, which is useful in dealing with missing data and does not require imputation [34]. Missingness was not associated with cannabis use, and therefore consistent with the assumption that outcome data were missing completely at random [35]. To account for missing data, we did adjust for employment, which was associated with attrition, in all models.

If perceptions of the health benefits of cannabis use increase over time and become more widespread (i.e., normative), cannabis use may increase further. Cannabis dependency may also increase, which has been linked to other substance use, depression and low satisfaction with life [36]. We found the number of days of cannabis use increased on average from 11 days pre-legalization to 13 days post-legalization, which may reflect movement toward cannabis abuse and dependency. A study in California found a link between the density of cannabis dispensaries and neighborhood ecology on cannabis abuse and dependency before the legalization of recreational cannabis [37]. Of interest is whether the density of dispensaries in California overall, and particularly in economically disadvantaged neighborhoods, occurred post-legalization and if cannabis use and dependency has risen disproportionately in some areas.

Further research examining the long-term effects of legalized recreational cannabis on health outcomes, perceptions, and use, especially among young people and those with depression and other mental illness, is warranted. Studies should also focus on the characteristics of cannabis used such as potency, as well as the environmental and social impacts. Public health communications and evidence-based interventions on the potential health benefits and consequences of cannabis use are needed.

## Conclusions

The current study provides novel insight into changes in reported cannabis use and health perceptions over time in relation to state policy change. Policy change in recreational cannabis use was associated with greater perceptions of health benefits of cannabis use over time. In a fully adjusted longitudinal model, factors related to cannabis use other than time were younger age, voting in favor of cannabis use, and positive health perceptions. Further research on the long-term patterns of health perceptions, exposures, and behaviors following the legalization of adult recreational cannabis use is warranted to inform future public health policies, interventions and educational initiatives.

## Data Availability

The datasets used and/or analysed during the current study can be requested from Dr. Ann Hsing, the PI of the Stanford WELL for Life Study, at annhsing@stanford.edu.

## Abbreviations

THC: Tetrahydrocannabinol
CBD: Cannabidiol
GEE: Generalized estimating eqs.
OR: Odds ratio
SD: Standard deviation
CI: Confidence interval
